# Prevalence and antimicrobial susceptibility patterns of *Shigella* among acute diarrheal outpatients in Mekelle hospital, Northern Ethiopia

**DOI:** 10.1186/s13104-015-1606-x

**Published:** 2015-10-28

**Authors:** Atsebaha Gebrekidan, Tsehaye Asmelash Dejene, Getahun Kahsay, Araya Gebreysus Wasihun

**Affiliations:** Department of Microbiology and Immunology, Institute of Biomedical Sciences, College of Health Sciences, Mekelle University, P.O. Box: 1871, Mekelle, Ethiopia

**Keywords:** *Shigella*, Drug resistance, Acute diarrhea, Outpatients, Mekelle hospital

## Abstract

**Background:**

Emergence of increased antimicrobial resistance of *Shigella* species is a global challenge, particularly in developing countries where increased misuse of antimicrobial agents occurs. There is no published data in the study area on the prevalence and antimicrobial susceptibility patterns of *Shigella* among acute diarrheal patients. This study was therefore, under taken to fill this gap.

**Methods:**

Using cross sectional study method, stool specimens were collected from 216 patients with acute diarrhea at Mekelle Hospital from August to November 2014. Standard bacteriological methods were used to isolate and determine the antimicrobial susceptibility patterns of the isolates, and data were analyzed using SPSS version 20.

**Results:**

Out of the total 216 participants, *Shigella* was isolated from 15 (6.9 %) of the participants. Ten (66.7 %) of the positive isolates were from children <15 years (p = 0.005). Latrine availability, source of drinking water and hand washing habits before meal were statistically significant with shigellosis (p < 0.05). Isolates of *Shigella* showed 100, 86.7 and 66.7 % resistance to amoxicillin, amoxicillin and cotrimoxazole respectively. Low levels of resistance were observed for norfloxacin and ciprofloxacin (6.7 % each). Overall, 80 % of the isolates showed multidrug resistance.

**Conclusion:**

*Shigella* isolates were highly resistant to amoxicillin, amoxicillin and cotrimoxazole. However, ciprofloxacin and norfloxacin were effective. Antibiotic surveillance is needed to prevent further emergence of drug resistant *Shigella* strains. More has to be done in the availability of latrine, supply of safe drinking water to the community to reduce the disease burden.

## Background

Shigellosis is the main cause of public health problem throughout the world. The disease is more common in resource limited countries. Though *Shigella* affects all, children under five are the most susceptible age group [1–3] as a result of poor personal hygiene [4], low immunity and lack of previous exposures [5]. More than 99 % of annual episodes of shigellosis with 1.1 million deaths have been reported from other developing countries [1]. Although the prevalence is less compared to developing nations, shigellosis is still a health issue in Europe [2] and United States [6].

Studies in Ethiopia showed that Shigellosis is the main cause of morbidity and mortality in children [7–15], mainly due to inadequate personal and environmental hygiene, their low immunity and limited access to safe drinking water [16, 17].

Extensive and uncontrolled prescription of antibiotics has led to the emergence of multi drug resistant *Shigella* strains [18]. This in turn has made it difficult in the selection of appropriate antibiotics [19] and effective treatment of shigellosis [20]. Empirical prescription of antibiotics by treating physicians is common in Ethiopia due to the lack of microbiological laboratory facilities to test antimicrobial susceptibility. As a result multi drug resistant strains of *Shigella* have been reported from different regions of the country [9–11, 14, 15].

Studies on shigellosis in Ethiopia were mostly focused on children, and most of them were retrospective type. The burden of the disease in adults seems to be overlooked. In contrary, there is no published data from the study area (Mekelle town) and the whole Tigray Regional State on the prevalence and antimicrobial susceptibility patterns of *Shigella.* Therefore, this study aimed to fill this gap by determining the prevalence and antimicrobial susceptibility patterns of *Shigella* in all age groups with acute diarrhea in Mekelle Hospital. This would complement the existing reports from other regions of country to help policy makers to have complete data and information as an input for further intervention programmes.

## Methods

### Study area, data collection and transportation

A cross sectional study was conducted in Mekelle general hospital from August 2014 to November 2014. Mekelle Hospital is the largest regional hospital serving about 6 million people residing in Tigray, Northern Amhara and Western Afar regions. The hospital is located in Mekelle city (the capital of Tigray National Regional State). Mekelle town is located 784 km North of Addis Ababa, the capital city of Ethiopia.

Consenting to participate in the study were obtained from patients and/guardians before by physicians. Consented participants were screened by physicians working in the medical and pediatric outpatient departments. Two hundred and sixteen stool specimens were collected from eligible patients using clean, sterile, wide-mouthed containers, free from disinfectant or detergent residue and tight-fitting leak-proof lids. Participants on antibiotic treatment at the time of sample collection and those with persistence diarrheal were excluded. Socio-demographic data were collected using questionnaire based interviews. Risk factors for acute diarrhea, and clinical signs and symptoms of patients were collected by clinicians during examination. Stool specimens were transported using Cary Blair transport media in cold box to Medical Microbiology laboratory of Mekelle University for further processing.

### Isolation and identification of *Shigella*

MacConkey agar, Xylose Lysine Deosxycholate agar and Selenite F enrichment broth (Oxoid, England) were used for isolation of *Shigella*. Culture negative specimens on primary solid media were sub-cultured from the enrichment broth to primary solid media to improve recovery of the isolates. All inoculated media were incubated at 37 °C for 18–24 h. After overnight incubation, non-lactose fermenters were further identified by biochemical tests using appropriate media namely: Kligler Iron Agar for carbohydrate fermentation test, Urea agar for the urea utilization test, tryptophan broth for Indole test, Simmon Citrate agar for citrate utilization, Motility agar for motility test, Lysine agar for lysine utilization test (all Oxoid, England).

### Antimicrobial susceptibility testing

Disk diffusion assay was performed to assess the antibiotic resistance/susceptibility pattern of the *Shigella* isolates. Antimicrobial susceptibility testing was carried out on Muller-Hinton agar (Oxoid, England) using the single disc diffusion technique against amoxicillin (10 μg), chloramphenicol (30 μg), co-trimoxazole (25 μg), ciprofloxacin (5 μg), norfloxacin (25 μg), amoxicillin (2 μg), amoxicillin clavulanic acid (30 μg) and gentamicin (10 μg) (Oxoid, England) based on the Standard Operating Procedure (SOP) adapted from Clinical and Laboratory Standards Institute (CLSI). Susceptibility results were reported as sensitive, intermediate and resistant. To standardize the inoculum density for a susceptibility test, BaSO_4_ turbidity standard, equivalent to a 0.5 McFarland standard was used by strictly following the SOP for the preparation and standardization [21]. An isolate was defined as multidrug resistant if it was resistant to three or more antimicrobial agents tested [22]. An isolate was defined as being multidrug resistant if it is resistant to three or more of the antimicrobial agents tested [20].

### Quality control and data analysis

A standard bacteriological procedure was followed to keep the quality of all laboratory tests. American Type Culture Collection (ATCC) strains (*Shigella sonni* ATCC 25331 and *Escherichia coli* ATCC 25922) were used as control strains for the culture and sensitivity testing. Data were entered and analyzed using SPSS version 20 for windows. Chi square test results were employed and p value less than 0.05 were considered significant.

### Ethical consideration

Ethical clearance was obtained from the Ethical Review Committee of Mekelle University, College of Health Sciences. Written permission was obtained from Tigray Regional Health Bureau and Mekelle Hospital and written consent was obtained from each participant (parent or guardian for children).

## Results

Out of the 216 participants, 109 (50.5 %) were male and 107 (49.5 %) were female. The age of the study participants ranged from 2 months to 80 years with a mean and a SD of 22.39 ± 18.02. Eighty (37 %) of the study participants were children up to 15 years old. From the total patients with acute diarrhea, 15 (6.9 %) were positive for *Shigella.* Children ≤15 years were more infected by shigella (P = 0.005, X^2^ = 11.52), but no *Shigella* was isolated from the age group of 31–45 years. Sex was not statistically significant with shigellosis in this study (P = 0.76, X^2^ = 0.093) (Table 1.)

**Table 1:** Prevalence of *Shigella* by age and gender among outpatients with acute diarrhea at Mekelle hospital, (August–November, 2014)

Characteristics	Total patients, N (%)	Positive isolates (%)	X^2^	*P* value
Gender				
Male	109 (50.9)	7 (6.4)	0.093	0.76
Female	107 (49.5)	8 (7.5)		
Age of patients (in years)				
≤15	80 (37)	10 (12.5)	11.52	0.005
16-30	82 (38)	2 (2.4)		
31–45	29 (13.4)	0 (0.0)		
≥46	25 (11.6)	3 (12)		

One hundred fifty (69.4 %) of the study participants visited the hospital 1–5 days after the onset of diarrhea. Ninety-three (43.1 %) and 78 (36.1) of the participants had mucoid and watery diarrhea respectively. Private toilet and pipe water source were available in 169 (78.2 %) and 154 (71.3 %) respectively. Absence of latrine at home (p = 0.002), source of drinking water (p = 0.027), and hand washing habit before meal (p = 0.029) were statistically significant with shigellosis. Eleven (8.6 %) of the positive patients reported no vomiting while abdominal cramp was manifested by 11 (8.1 %) of the positive patients (Table 2).

**Table 2:** Prevalence of *Shigella* by clinical symptoms and risk factors among acute diarrhea patients at Mekelle hospital (August to November, 2014)

Characteristics	Patients, N (%)	Positive isolates (%)	X^2^	P value
Fever				
Yes	87 (40.3)	7 (8)	NA	NA
No	129 (59.7)	8 (6.2)		
Vomiting				
Yes	88 (40.7)	4 (4.5)	NA	NA
No	128 (59.3)	11 (8.6)		
Abdominal cramp				
Yes	136 (63)	11 (8.1)	NA	NA
No	80 (37)	4 (5)		
Duration of diarrhea				
1–5 days	150 (69.4)	6 (4)	NA	NA
6–10 days	34 (15.7)	4 (11.8)		
11–14 days	32 (14.8)	5 (15.6)		
Consistency of diarrhea				
Watery	78 (36.1)	5 (6.4)	NA	NA
Mucus	93 (43.1)	2 (2.2)		
Bloody	19 (8.8)	4 (21.1)		
Mucus + blood	26 (12)	4 (15.4)		
Presence of latrine at home				
Yes	169 (78.2)	7 (4.1)		
No	47 (21.8)	8 (17)	7.5	0.002
Source of drinking water				
Private pipe water	154 (71.3)	6 (3.9)		
Public pipe water	37 (17.1)	6 (16.2)		
Well	25 (11.6)	3 (12)	9.19	0.027
Hand washing habit before meal				
Yes	154 (71.3)	7 (4.5)		
No	62 (28.7)	8 (12.9)	4.78	0.029
Hand washing habit				
With soap	36 (23.4)	1 (2.8)		
Without soap	118 (76.6)	6 (5.1)	0.338	0.561

*NA* not applicable as they are not predisposing factors

**Fig. 1:**
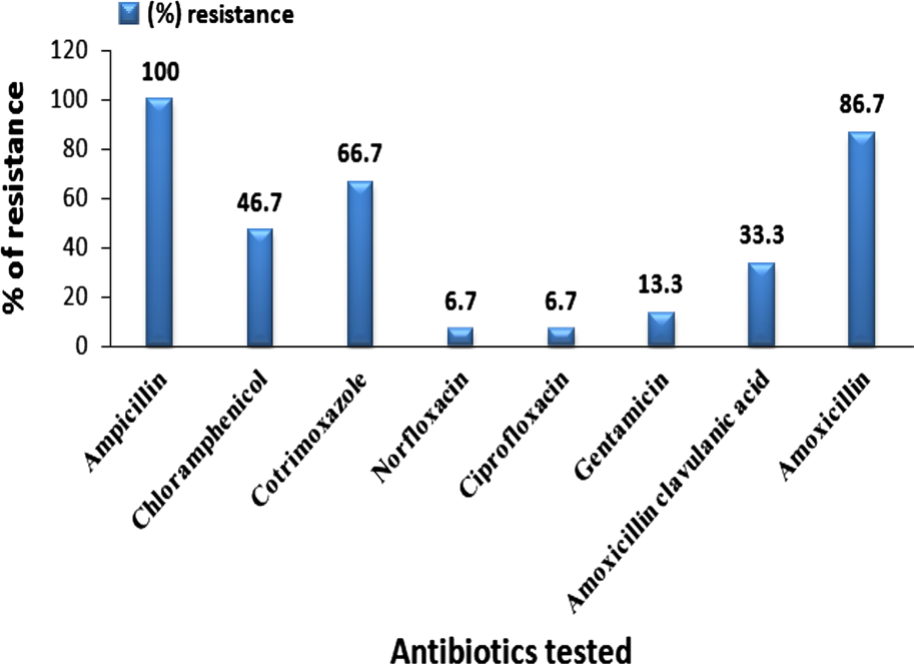
Antimicrobial resistance patterns of *Shigella* isolates from diarrheic outpatients at Mekelle hospital (August to November, 2014)

Antimicrobial susceptibility pattern in this study showed that *Shigella* isolates were highly resistant to amoxicillin (100 %), amoxicillin (86.7 %) and cotrimoxazole (66.6 %). Low resistance was observed to ciprofloxacin and norfloxacin (6.7 % each), gentamicin (13.3 %) and amoxicillin clavulanic acid (33.3 %) (Fig. 1).

Antibiogram pattern in this study revealed that 12 (80 %) of the isolates were multidrug resistance (resistance for more than two antibiotics) while none of them was sensitive to all antimicrobial drugs tested. Four (26.7 %) of the *Shigella* isolates have developed resistance to three antimicrobials. Eight of the isolates were resistant to 4 antibiotics and one isolates was resistant to five, one for six antibiotics (Table 3).

**Table 3:** Multidrug resistance pattern of *Shigella* isolates from outpatients with acute diarrhea at Mekelle hospital (September to November, 2014)

Number of antimicrobial resistance	*Shigella* species	
	Resistance antibiogram^a^	Number of isolates (%)
Ro	None	None
R1	AMP	1 (6.7)
R2	AMP, AML	1 (6.7)
	AMP, AMC	1 (6.7)
R3	AMP, C, AML	1 (6.7)
	AMP, AML, SXT	1 (6.7)
R4	AMP, C, AML, SXT	4 (26.7)
	AMP, AML, AMC, SXT	3 (20)
	AMP, C, AML, AMC	1 (6.7)
R5	AMP, AML, SXT, CIP, CN	1 (6.7)
R6	AMP, C, AML, SXT, NOR, CN	1 (6.7)

*AMP* amoxicillin, *C* chloramphenicol, *AML* amoxicillin, *AMC* amoxicillin clavulanic acid, *SXT* sulphamethoxazole trimethoprim, *NOR* norfloxacin, *CIP* ciprofloxacin, *CN* gentamicin

^a^Ro, R1,R2,R3,R4,R5,R6 = Sensitive to all, resistant to one, two, three, four, five and six antibiotics respectively

There is a gradual decreasing of *Shigella* prevalence in the country over time; though it is not uniform in all parts of the study areas. However, drug resistant level of *Shigella* isolates is increasing especially for the orally administered antimicrobials treatments (Table 4).

**Table 4:** Pattern of prevalence and antimicrobial resistance patterns of *Shigella* reported from 2001 to 2014 in Ethiopia

Study area	Authors	Prevalence	Amp	Cot	C	NOR	CIP	CN	AMC	AMX
Gondar	Yismaw et al. [9]	7.4	79.9	73.4	52.2	–	8.9	7.9	–	–
	Huruy et al. [10]	16.8	81.5	75.4	50.8	–	9.2	10.7	–	–
	Tiruneh [11]	7.5	78.9	84.6	67.8	1.1	2.2	12.2	–	–
	Demissie et al. [12]	4.57	94.1	58.8	17.6	–	0.0	41.7	–	–
Harar	Reda et al. [14]	6.7	100	5.9	29.4	–	–	0.0	–	100
Bahir Dar	Debas et al. [15]	14.5	–	–	–	9.4	0.0	25	–	88.2
Hawassa	Mulatu et al. [13]	7	63	0.0	9.1	–	0.0	27.3	–	100
Butajira	Mengstu et al. [29]	4.5	47.1	76.5	29.4	–	5.9	17.6	–	–
Jimma	Mache [7]	20.1	70.1	32.5	40.3			1.3	–	–
	Beyene and Tasew [8]	2.3	100	100	16.7	–	–	0.0	–	100
Mekelle	This study	6.9	100	66.7	46.7	6.7	6.7	13.3	33.3	86.7

*AMP* amoxicillin, *C* chloramphenicol, *AML* amoxycillin, *AMC* amoxycillin Clavulanic acid, *SXT* sulphamethoxazole trimethoprim, *NOR* norfloxacin, *CIP* ciprofloxacin, *CN* gentamicin

## Discussion

The isolation rate of Shigella (6.9 %) in our study was comparable to previous studies in Ethiopia (Gondar,7.4 % [11], Harar 6.4 % [14] Hawassa 7 % [13], Addis Ababa 5 % [23]) and other countries, Nigeria, 7.7 % [24] Trinidad 8 % [5], Iran 8.8 % [25] and Western Nepal 6.88 % [26].

Our prevalence was however; lower when compared to studies conducted in other parts of Ethiopia (Jimma, 20.1 % [7] Gondar 16.8 % [10] and Bahir Dar,14.5 % [15] ), Nepal 13.61 % [27] and India 12.1 % [19]. Our prevalence was slightly higher than studies conducted from Ethiopia: Jimma, 2.3 % [8] Gondar, 4.57 % [12] and Butajira, 4.5 % [28]^.^ Differences insanitation and personal hygiene, access to safe drinking water and methodology (sample size, study participants, study design) may be the possible reasons for the variation in the prevalence.

The variation in the prevalence of *Shigella* over time in the study area could not be detailed in this study due to the absence of previous studies from Mekelle town and Tigray regional state. However, the pattern of shigellosis in other parts of Ethiopia has shown a decreasing trend in prevalence overtime though the decrement is not uniform. That could be due to the improved awareness of the community about personal and environmental hygiene from the continuous interventions made by the health extension workers implemented by the Ethiopian government, and improved supply of safe drinking water.

Although majority of the study participants recruited were 16–45 years of age, only 2 (2.4 %) were positive for *Shigella*. Higher positivity for *Shigella* was recorded from children up to 15 years. This was similar to the report from Gondar [11]. This may be due to their substandard personal hygiene, low immune resistance and more exposure to unavailability of safe water for drinking and washing hands in school compounds.

About two-third of the study participants came to the hospital 1–5 days after the onset of diarrhea yet, high rates of *Shigella* species were found among study participants diarrhea who reported 11–14 days after their onset of diarrhea. This long time before seeking health services may contribute for the spread of the pathogen in the community.

Abdominal pain, vomiting and fever were the predominant symptoms of culture positive *Shigella* cases in this study. Similar results have been reported from other studies [29]. This is due to the ability of the bacteria to invade and replicate in cells lining the colon and rectum, patients with bloody diarrhea and mixed (mucus and blood) in this study were more positive to *Shigella*. This was in contrary to results from other parts of Ethiopia [13, 28]. This difference may result from differences of the species involved: *Shigella dysentery* and *Shigella sonnei* cause bloody and watery diarrhea respectively. Absence of latrine at home, source of drinking water, failure to wash hands before meals were found significantly associated with shigellosis; which is similar with reports from elsewhere [13, 14, 19]

Antimicrobial resistance pattern and prevalence of *Shigella* in this study are compared with previous findings from other parts of the country are shown in Table 4. The resistance patterns of antimicrobial drugs to *Shigella* in the present study ranges from amoxicillin (100 %) to ciprofloxacin and norfloxacin (6.7 % each). The resistance pattern amoxicillin in this study was similar with studies carried out in other regions of Ethiopia (Jimma [8] and Harar [14]), India [19] and Iran [20]. The rise in resistance may be due to ease of availability and repeated use for many years.

In this study (13.3 %) of the isolates were Gentamicin resistant; this was similar to the study done in Gonder (12.2 %) [18] and Butajira (17.6 %) [28]. However, results obtained from Addis Ababa [30], and Harar [14] showed no resistance to Gentamicin. Unlike to our results, however; relatively high resistant isolates for gentamicin were recorded from Bahir Dar (25 %) [15], Hawassa (27.3 %) [13] and Gondar (41.7 %) [12]. This indicates emerging of gentamicin drug resistance *Shigella* isolates over time. This was clearly seen in Gonder where gentamicin resistance is increasing from 7.9 % in 2006 9 to 41.7 % in 2014 [12].

Low level *Shigella* resistance to ciprofloxacin 6.7 % were observed in this study similar to studies from Ethiopia (Bahir Dar (0.0 %) [15], Hawassa (0.0 %) [13] and Gondar (0.0 %) [12], Brazil (0.0 %), [31] Iran (0.0 %), [20] Ghana (0.0 %) [32]. However, results from other countries showed high resistance to ciprofloxacin: India (82 %), [19] Western Nepal 47.8 % [26], Nepal 28.3 % [33] and China 25.2 % [32]). High resistance (86.7 %) of *Shigella* was also seen to amoxicillin in this study which was similar to the report from Bahir Dar 88.2 % [15]^.^However, higher resistance than this study were reported from Hawassa 100 % [13], Jimma 100 % [7] and Harar 100 % [14] which could be due to the variation in clinicians’ prescription of the antibiotic for the treatment of patients.

High resistance was also observed to co-trimoxazole 66.7 %, which agrees with the reports from Gonder 73.4 % [10], North West Ethiopia 84.6 % [11], Butajira 76.5 % [28], Jimma 100 % [7] in contrast to low resistance report from Hawassa (56.0 %) [13].This increase of resistance from those reports indicated that aggravating problem of drug resistance by these microbes over the years. This may be due to misuse or inappropriate use of drugs.

Anti biogram patterns revealed that none of the *Shigella* isolates in the present study were sensitive to all tested antibiotics. However, 80 % of the isolates showed multidrug resistance (resistance for more than two antibiotics) (Table 3). This shows that even if antibiotics have revolutionized the treatment of common bacterial infections and played a crucial role in reducing mortality, there is rapid increase in antibiotic resistance among *Shigella* pathogens in developing countries which needs is critical attention.

The overuse and misuse of antibiotics in the treatment of diarrhea could lead to an increase of antibiotic resistance [14, 34]. Limited laboratory diagnosis in developing countries imposes clinicians to syndromaic diagnosis and empirical prescription of broad spectrum antibiotics that led drug resistant bacterial strains to emerge [35]^.^

The strength of this study compared to previous studies on *Shigella* is in the design of the study. Our study was conducted prospectively in a manner of controlled data collection and laboratory tests, whereas the other studies were conducted retrospectively: Awassa [13], Gondar [9, 11] Jimma [7] and Addis Ababa [30]. This study may not necessarily be representative of the community prevalence of the disease, because the sample size is small.

## Conclusion

Shigellosis in this study was 6.9 %. Children under 15 years were highly infected. Source of drinking water, presence of latrine in their compound and hand washing habit before meal were found as risk factors. *Shigella* isolates were high resistant to amoxicillin 100 and 66 % cotrimoxazole. Ciprofloxacin, norfloxacin and gentamicin were found highly sensitive for *Shigella* isolates. More emphasis should be given towards supply of safe water and health education for the community. Accurate diagnosis during management of infection caused by Shigella should be employed than empirical treatment of patients. Periodic epidemiological surveillance is of great importance to control the diseases and MDR of *Shigella* spp.
